# LightCpG: a multi-view CpG sites detection on single-cell whole genome sequence data

**DOI:** 10.1186/s12864-019-5654-9

**Published:** 2019-04-23

**Authors:** Limin Jiang, Chongqing Wang, Jijun Tang, Fei Guo

**Affiliations:** 1 School of Computer Science and Technology, College of Intelligence and Computing, Tianjin University, Tianjin, China; 2 School of Chemical Engineering and Technology, Tianjin University, Tianjin, China; 3 Department of Computer Science and Engineering, University of South Carolina, Columbia, SC, USA

**Keywords:** DNA methylation, Positional features, Structural features, Sequence features, LightGBM

## Abstract

**Background:**

DNA methylation plays an important role in multiple biological processes that are closely related to human health. The study of DNA methylation can provide an insight into the mechanism behind human health and can also have a positive effect on the assessment of human health status. However, the available sequencing technology is limited by incomplete CpG coverage. Therefore, it is crucial to discover an efficient and convenient method capable of distinguishing between the states of CpG sites. Previous studies focused on identifying methylation states of the CpG sites in single cell, which only evaluated sequence information or structural information.

**Results:**

In this paper, we propose a novel model, LightCpG, which combines the positional features with the sequence and structural features to provide information on the CpG sites at two stages. Next, we used the LightGBM model for training of the CpG site identification, and further utilized sample extraction and merged features to reduce the training time. Our results indicate that our method achieves outstanding performance in recognition of DNA methylation. The average AUC values of our method using the 25 human hepatocellular carcinoma cells (HCC) cell datasets and six human heptoplastoma-derived (HepG2) cell datasets were 0.9616 and 0.9213, respectively. Moreover, the average training times for our method on the HCC and HepG2 datasets were 8.3 and 5.06 s, respectively. Furthermore, the computational complexity of our model was much lower compared with other available methods that detect methylation states of the CpG sites.

**Conclusions:**

In summary, LightCpG is an accurate model for identifying the DNA methylation status of CpG sites in single cells. Furthermore, three types of feature extraction methods and two strategies used in LightCpG are helpful for other prediction problems.

**Electronic supplementary material:**

The online version of this article (10.1186/s12864-019-5654-9) contains supplementary material, which is available to authorized users.

## Background

DNA methylation is a topic of much debate in the epigenetic world, but understanding of DNA methylation has great room to upgrade [1, 2]. One of the most common ways for identifying DNA methylation is identifying the cytosine-5 methylation within the CpG dinucleotides [3]. DNA methylation can affect the functional state of regulatory regions and affect DNA replication and gene transcription. These functions are closely related to many human diseases, including malignant tumors, immune diseases, and Alzheimer’s [4–7]. Recent studies have found that methylation levels are closely related to age and can, therefore, be indicative of life expectancy [8]. Specifically, previous studies pointed that DNA methylation levels change with age [9, 10]. Deary et al. [11] identified that in the elderly population, if the estimated DNA methylation level age has five years higher than the actual age, it came along that the risk of death will increase by 21 percent. Therefore, the study of DNA methylation has important clinical and medical significance. Traditional methods evaluating methylation sites include bisulfite genomic sequence (BGS), methylation-specific PCR (MSP), and high-resolution melting (HRM), which are time-consuming and expensive. Therefore, using more efficient computational methods to identify DNA methylation is very important and is also critical to making methylation predictions more reliable [12].

The characteristics of methylation sites are inevitably digitized when using computational methods that identify them. Many previous studies [13–16] have demonstrated that the sequence of neighboring nucleotides of one methylation site is specific and that the methylation state is closely related to the sequence information, which allows for the prediction of the methylation state only based on the sequence composition. The Methylator method [14] proposed by Bhasin et al. used conventional binary sparse encoding to directly convert sequences into a feature vector. The method described by Das et al. [17] involves extracting a sequence with the window size of 800 bp, counting the methylation propensity, and using the principal component analysis (PCA) with recursive feature elimination for feature selection. Recently, Pan et al. [18] employed an n-gram, multivariate mutual information [19], Discrete Wavelet Transform [20] and Pseudo Amino Acid Composition [21] to extract DNA sequence features with a window size of 100 bp.

With the discovery of various biological processes, methylation is found to be closely related to many proteins [22, 23]. Therefore, the structural information of the protein can be used for the identification and better profiling of methylation sites. Structural features discussed by Bock et al. [24] included the frequency and distribution of CpG islands (CGIs), exon distribution, transcription factor binding sites (TFBS), and single nucleotide polymorphisms (SNP), with a total of 918 features representing the properties of the CpG sites. Zhang et al. [25] extracted a total of 841 features including histone modification features and then used PCA to select features for downstream CpG site identification. Fan et al. [26] extracted four histone methylation marks to identify CpG sites. Zhang et al. [1] extracted genomic positional features, neighbor features, sequence properties, and cis-regulatory elements to identify CpG sites. Saif et al. [27] identified highly methylated regions using promoter region information.

Following the feature extraction at CpG sites, it is important to select an appropriate model for CpG site identification. Most of the previous methods used support vector machine (SVM) as the classification model, which resulted in an excellent performance and creation of tools such as The Methylator [14] and HDFINDER [17]. Moreover, additional methods using random forest (RF) [28] have also achieved excellent results. Furthermore, the method by Pan et al. used sparse Bayesian learning model [29]and also achieved good performance results. Therefore, the selection of the appropriate classifier can directly affect the model performance.

In the methods discussed above, the performance of single-cell methylation state prediction can be affected by the density of the sites measured in the dataset. Recently, researchers have developed several single-cell DNA methylation group sequencing methods, such as single-cell bisulfite sequencing (scBS-seq) [30] and single-cell reduced-representation bisulfite sequencing (scRRBS-seq). Smallwood et al. [31] discovered that the CpG coverage of the scBS-seq method is only 20−40*%* and that of the scRRBS-seq method is only 1−10*%* [32–34]. It is important to note that the decrease in coverage may result in a loss of information. Therefore, the key focus is to determine the state of the missing CpG sites in the entire genome. The methods cited above, which use sequence and structural features can only resolve methylation state prediction at different sites within a single cell and cannot account for associations between multiple cells. Therefore, these methods are not suitable for the examination of methylation states in multiple cells. The DeepCpG model, proposed by Christof et al. [35], used 25 CpG sites upstream and downstream of different sites in different cells, and used the site state, distance between each site and target site as features. This method allowed for the connection between various cells through the use of the deep learning model gated recurrent network (GRU), and also extracted features from the DNA sequence by convolutional neural network (CNN) and a fully connected hidden layer. Next, the use of the DeepCpG fully connected the deep learning to identify CpG sites and achieved an impeccable accuracy. However, the DeepCpG model utilizes a large amount of time during the training process.

Inspired by the DeepCpG model, we posit that some of the same CpG sites with unknown methylation states can be detected in multiple cells, and that the states of these sites can vary between different cells. We extracted the CpG site information as novel positional features to build the model. Importantly, we used three-part feature approach (sequence features, structural features, and novel positional features) to identify the multi-cell CpG sites. Moreover, we produced the sparse binary features, such as most of the structural features and half of the positional features. Finally, we constructed the CpG recognition model using the LightGBM model [36]. Experiments demonstrate that our method can predict the states of missing CpG sites in multiple cells with high precision and efficiency.

## Methods

**Fig. 1:**
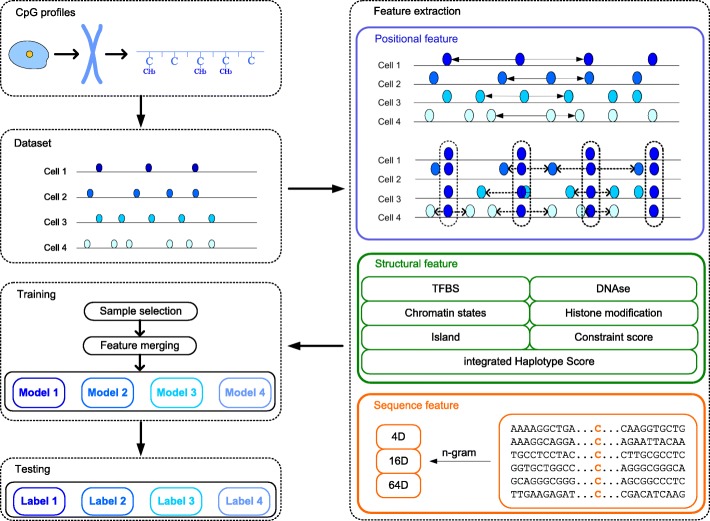
The flow chart of LightCpG. **CpG** **profiles** are obtained from scTrio-seq. **Dataset** includes multiple single-cell CpG profiles. **Feature** **extraction**: positional feature includes methylation state and the distance between the sites; structural feature includes CpG islands (CGIs) status (CGIs, CGIs shore, CGIs shelf), cis-regulatory elements (TFBS, DNase, chromatin states, histone modification), and DNA properties (integrated haplotype score (iHS), constrain score); sequence feature includes 84 dimension features that are extracted using DNA sequence and n-gram method. **Training**: LightGBM is used to construct a model for each single-cell CpG data; sample selection is used to reduce the number of samples; feature merging is used to reduce the number of features. **Testing**: the trained LightCpG model can be used for prediction of the new CpG sites

In this paper, we propose a novel method to resolve the issue of methylation identification, as shown in Fig. 1. First, we extracted sequence features, structural features and positional features of known CpG sites. Then, we applied the LightGBM model to train the classifier for each cell and also adjusted the model parameters to get the best performance. Finally, we used our trained model, called LightCpG, to predict the methylation states of unknown CpG sites.

### Dataset

We downloaded two benchmark datasets Homo sapiens GM12878 (ENCFF001TLS) and heart left ventricle (ENCFF001TKC), which were extracted by reduced representation bisulfite sequencing (RRBS) from ENCODE [37, 38]. Single-cell triple omics sequencing (scTrio-seq) is a sparse single-cell CpG profile. We downloaded two datasets of scTrio-seq profiled cells, 26 human hepatocellular carcinoma cells (HCCs) cells and six human heptoplastoma-derived (HepG2) cells, from the gene expression omnibus (GSE65364). Based on the study by Hou et al. [34], the Ca26 was excluded because the distribution of methylation state is seriously abnormal, so there were only 25 cells in the HCCs cell dataset. Every position of CpG sites was mapped to hg19 by using the liftOver tool (http://www.genome.ucsc.edu/cgi-bin/hgLiftOver) from the UCSC Genome Browser [39]. In this paper, we examined these sites as research objects, which were covered by at least four reads.

Inspired by the DeepCpG [35], we adopted the same validation method for all datasets. In the experimental part, the CpG sites in the training set were from chromosomes 1, 3, 5, 7, 9, and 11, and those in the test set were from chromosomes 2, 4, 6, 8, 10, and 12, and finally those in the validation set were from chromosomes 13, 14, 15, 16, 17, 18, and 19. All datasets are described in detail in Additional file 1 and Additional file 2.

### Feature extraction

#### Structural feature

In the mammalian genome, the CGIs are specific regions where the density of unmethylated CpG sites is greater compared to other regions. Work by Zhang et al. [1] demonstrated that the methylation level in CGIs is below 50%, the methylation level in CGIs shores ranges between 20% and 80% and the methylation level in CGIs shelves is much higher compared to the average level. Therefore, all samples in this paper were assigned three binary features. Specifically, the value of CGIs feature was 1 if the sample sites were within CGIs regions, otherwise it was 0. We applied the same principles to the CGIs shore feature and the CGIs shelf feature. These parts of the data were downloaded from the UCSC genome browser [40].

Studies indicate that CGIs have been shown to co-localize with the DNA regulatory elements, including TFBS, histone modification marks, chromatin states and DNase I hypersensitive sites (DHSs) [1, 41]. Moreover, many studies have found that the methylation states of CGIs are closely related to TFBSs [42–44]. DNA methylation and histone modifications are involved in regulating gene repression patterns during cell development as demonstrated by traditional experiments [45]. Some studies found that chromatin modification and DNA methylation are mutually dependent in the aspect of gene regulation [46]. Moreover, DHSs are linked with a strong enrichment of CpG methylation [47, 48]. These feature data were downloaded from the ENCODE [49]. All of the above-referenced features were binary.

Importantly, DNA methylation, an important epigenetic modification, is one of the major mechanisms regulating gene expression during cell differentiation. Although it does not change the genetic sequence, it can be inherited by offspring. Therefore, we used the integrated haplotype score (iHS) http://hgdp.uchicago.edu/Browser_tracks/iHS and the GERP++ constraint score on hg19 [50] http://mendel.stanford.edu/SidowLab/downloads/gerp/ to recognize the CpG sites in the DNA sequence.

Overall, we obtained 175 structural features for each CpG site, including 144 specific TFBSs, 15 chromatin states, 10 histone modification marks, CGIs, CGIs shores and shelves, DHSs, iHSs, and constraint scores.

#### Sequence feature

According to the position of the CpG sites in the raw data files, we extracted the sequence from the reference hg19, including the extracted DNA sequence of 101 bp with 50 bp before and 50 bp after the CpG site. The DNA primary sequence is composed of adenine (A), thymine (T), cytosine (C) and guanine (G). Some studies [51] suggest that the primary sequence composition is critical for the methylation recognition.

In this paper, we use DNA sequence information extracted by *n*-gram to identify the CpG sites. Each feature of *n*-gram can be denoted as a pair of value (*v*_*i*_,*f*_*i*_), where *v*_*i*_ is one feature that can be recorded as a combination of *n* nucleotides and *f*_*i*_ represents the frequency of *v*_*i*_ in the DNA sequence. The equation for *f*_*i*_ is shown below:

1 $$  f_{i} = \frac{N(v_{i})}{L-(n-1)},  $$

where *N*(*v*_*i*_) represents the number of *v*_*i*_ in the DNA sequence; *L* represents the length of the DNA sequence and *n* represents the number of nucleotides in the *v*_*i*_.

In this paper, we use 1/2/3-gram to represent one DNA sequence, denoted as *v*_*i*_∈{*A,C*,*T,G*}∪{*AA,AC*,…,*GT,GG*}∪{*AAA,AAA*,…,*GGT,GGG*}. Finally, we used 84 sequence features to identify the CpG sites

#### Positional feature

**Fig. 2:**
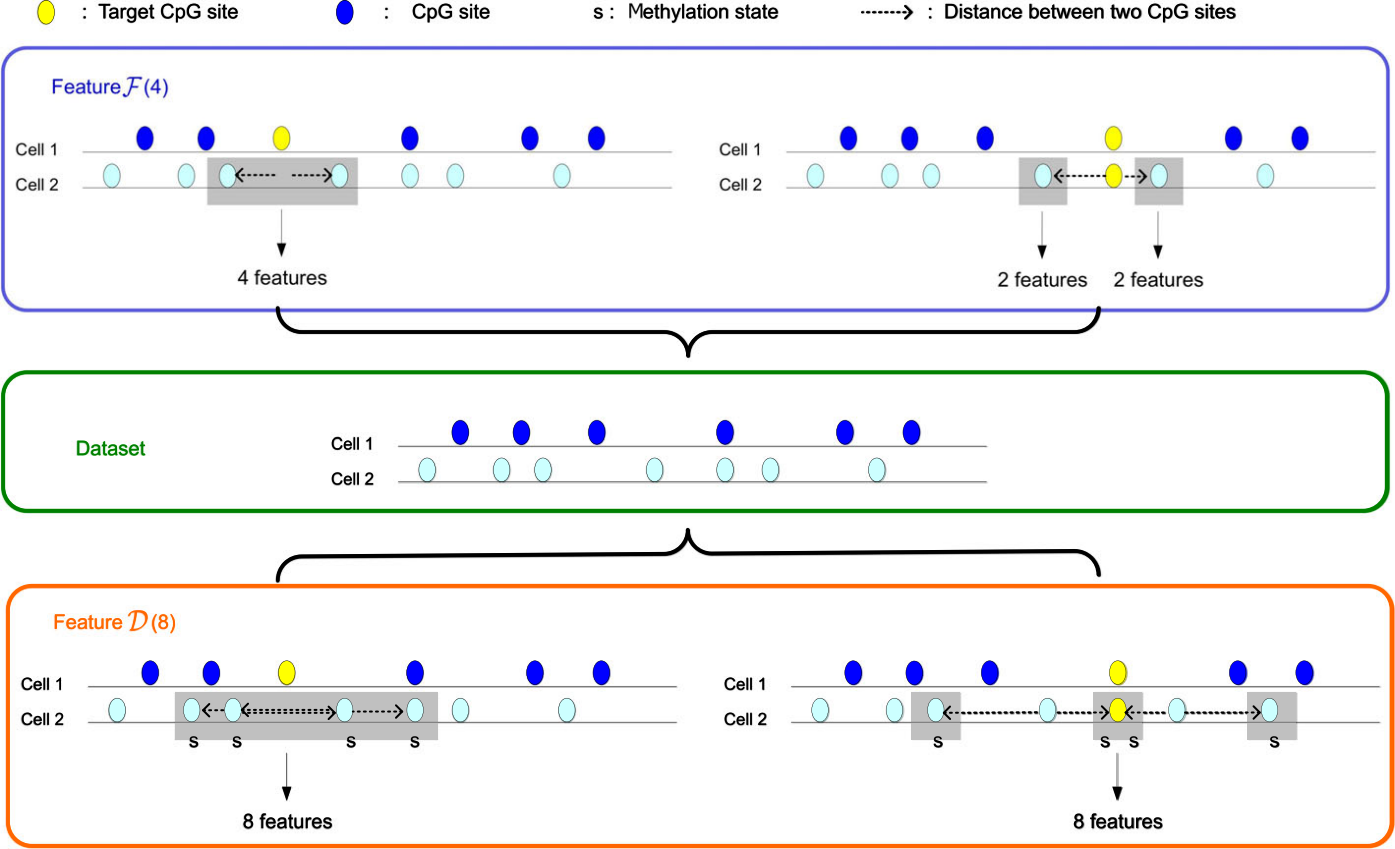
The sketch map of skip-*k* method

In this section, we extracted positional features by using the information on the CpG states and the distance between the adjacent CpG sites. We proposed a novel skip-*k* method to analyze the correlation between two adjacent CpG sites for DNA methylation recognition. The skip-*k* method is composed of two parts: skip- *k*_1_ and skip- *k*_2_. The sketch map of skip-*k* is shown in Fig. 2.

The correlation between two adjacent CpG sites can be verified by skip- *k*_1_. The method skip- *k*_1_ separately extracts one CpG site at the *k*_1_-th distance upstream and downstream of the target CpG site, and then extracts the states of these two CpG sites and calculates the distances between them and the target CpG site. These two states and two distances are used to predict the target CpG sites. Next, we analyzed the change in prediction accuracy in the different *k*_1_ values. Specifically, we employed two benchmark datasets (GM12878 and heart left ventricle) to discuss the skip- *k*_1_ method. The experimental results demonstrate that the correlation between two CpG sites became weaker as the distance between them increased.

The correlation between CpG sites in a specific window can be analyzed by skip- *k*_2_. The skip- *k*_2_ method separately extracts the *k*_2_ nearest the CpG sites from upstream and downstream of the target CpG site, and then separately extracts the states of these CpG sites and calculates the distances between them and the target CpG site. These 2*k*_2_ states and 2*k*_2_ distances are used to distinguish the state of the target CpG site. We analyzed the change in prediction accuracy with the different windows. Next, we used the 25 cells of the human HCCs dataset to analyze the skip- *k*_2_ method. The experimental results demonstrate that the prediction accuracy became smaller as the length of window increased.

Therefore, in this paper, both the *k*_1_ and *k*_2_ values were set as 1. We separately extracted one CpG site in the vicinity (upstream and downstream) of the target CpG site and then extracted the states of these two CpG sites and calculated the distances between them and the target CpG site. These four features were used to predict the CpG sites. For multiple human cell line cells, Christof et al. [35] established a bidirectional GRU model that builds the connection between multiple cells using the window length *k*_2_=25. This model achieved excellent performance based on five datasets. However, in the modeling process, the model only considered the methylation states and the distances of adjacent sites in the window for each cell, ignoring some information about the methylation states of the same sites in different cells.

In this section, we extracted the features of the same CpG sites in other cells. In different cells, some of the same CpG sites have unknown methylation states. We solved the problem of feature extraction at the same CpG sites in different cells. Assuming that there are *m* cells in the data, we defined a set *G*_*i*_ that contains *n* CpG sites on each chromosome in the *i*-th cell, denoted as follows:

2 $$ \begin{aligned} G_{i} &= \left\{\left(p_{i}^{1},s_{i}^{1}\right),\left(p_{i}^{2},s_{i}^{2}\right),\ldots,\left(p_{i}^{c},s_{i}^{c}\right),\ldots\left(p_{i}^{n},s_{i}^{n}\right)\right\}; \\& \quad i=1,2,\ldots,m \end{aligned}  $$

where $p_{i}^{c}$ and $s_{i}^{c}$ represent the position and methylation state of the *c*-th CpG site in the *i*-th cell, respectively.

To maximize the features for the same sites in different cells, we use *G*_*i*_ and *G*_*j*_ to establish the following feature extraction method. If one CpG site existed in the *j*-th cell satisfying $p_{i}^{c}=p_{j}^{l}$, we denoted $\mathcal {F}_{i,j}^{c}$ as the distance and the methylation states of the nearest CpG sites on both sides of the *l*-th CpG site in the *j*-th cell, as shown below:

3 $$  \mathcal{F}_{i,j}^{c} = \left\{P_{i,j}^{l-1},S_{i,j}^{l-1},P_{i,j}^{l+1},S_{i,j}^{l+1}\right\}  $$

where $P_{i,j}^{l-1}=p_{j}^{l-1}-p_{i}^{c}, P_{i,j}^{l+1}=p_{j}^{l+1}-p_{i}^{c}, S_{i,j}^{l-1}=s_{j}^{l-1}$ and $S_{i,j}^{l+1}=s_{j}^{l+1}$.

If one CpG site is unknown methylation status in the *j*-th cell, we selected two neighboring CpG sites in the *j*-th cell satisfying $p_{j}^{l} < p_{i}^{c} < p_{j}^{l+1}$, following which $\mathcal {F}_{i,j}^{c}$ was denoted as shown below:

4 $$  \mathcal{F}_{i,j}^{c} = \left\{P_{i,j}^{l},S_{i,j}^{l},P_{i,j}^{l+1},S_{i,j}^{l+1}\right\}  $$

where $P_{i,j}^{l}=p_{j}^{l}-p_{i}^{c}, P_{i,j}^{l+1}=p_{j}^{l+1}-p_{i}^{c}, S_{i,j}^{l}=s_{j}^{l}$ and $S_{i,j}^{l+1}=s_{j}^{l+1}$.

Furthermore, we represented the features for the same CpG sites in different cells and established the following feature extraction method. If one CpG site existed in the *j*-th cell satisfying $p_{i}^{c}=p_{j}^{l}$ and *i*≠*j*, we denoted $\mathcal {D}_{i,j}^{c}$ as the distance and the methylation states of the nearest CpG sites on both sides of the (*l*−1)-th and (*l*+1)-th CpG sites in the *j*-th cell, as shown below:

5 $$ \begin{aligned} \mathcal{D}_{i,j}^{c} = &\left\{P_{j,j}^{(l-2)(l-1)},S_{j,j}^{l-2},P_{j,j}^{(l-1)(l)},S_{j,j}^{l}\right\}\\ \cup&\left\{P_{j,j}^{(l)(l+1)},S_{j,j}^{l},P_{j,j}^{(l+1)(l+2)},S_{j,j}^{l+2}\right\} \end{aligned}  $$

where $P_{j,j}^{(l-2)(l-1)}=p_{j}^{l-1}-p_{j}^{l-2}, S_{j,j}^{l-2}=s_{j}^{l-2}, P_{j,j}^{(l-1)(l)}=p_{j}^{l}-p_{j}^{l-1}, S_{j,j}^{l}=s_{j}^{l}, P_{j,j}^{(l)(l+1)}=p_{j}^{l+1}-p_{j}^{l}$ and $P_{j,j}^{(l+1)(l+2)}=p_{j}^{l+2}-p_{j}^{l+1}, S_{j,j}^{l+2}=s_{j}^{l+2}$.

These features included the methylation states of the same sites in different cells. If one CpG site is unknown methylation status in the *j*-th cell, we selected two neighboring CpG sites in the *j*-th cell satisfying $p_{j}^{l} < p_{i}^{c} < p_{j}^{l+1}$, and then $\mathcal {D}_{i,j}^{c}$ was denoted as shown below:

6 $$ \begin{aligned}  \mathcal{D}_{i,j}^{c} = &\left\{P_{j,j}^{(l-1)(l)},S_{j,j}^{l-1},P_{j,j}^{(l)(l+1)},S_{j,j}^{l+1}\right\}\\ \cup&\left\{P_{j,j}^{(l)(l+1)},S_{j,j}^{l},P_{j,j}^{(l+1)(l+2)},S_{j,j}^{l+2}\right\} \end{aligned}  $$

where $P_{j,j}^{(l-1)(l)}=p_{j}^{l}-p_{j}^{l-1}, S_{j,j}^{l-1}=s_{j}^{l-1}, P_{j,j}^{(l)(l+1)}=p_{j}^{l+1}-p_{j}^{l}, S_{j,j}^{l+1}=s_{j}^{l+1}, S_{j,j}^{l}=s_{j}^{l}, P_{j,j}^{(l+1)(l+2)}=p_{j}^{l+2}-p_{j}^{l+1}$ and $S_{j,j}^{l+2}=s_{j}^{l+2}$.

If $i=j, \mathcal {F}_{i,i}^{c}$ are positional features in the same cell. $\mathcal {D}_{i,i}^{c} = \left \{P_{i,i}^{l-2},S_{i,i}^{l-2},P_{i,i}^{l},S_{i,i}^{l},P_{i,i}^{l},S_{i,i}^{l},P_{i,i}^{l+2},S_{i,i}^{l+2}\right \}$, which contains the methylation state of the $p_{i}^{c}$ site. Therefore, $\mathcal {D}_{i,i}^{c}$ must avoid the condition of *i*=*j*.

**Fig. 3:**
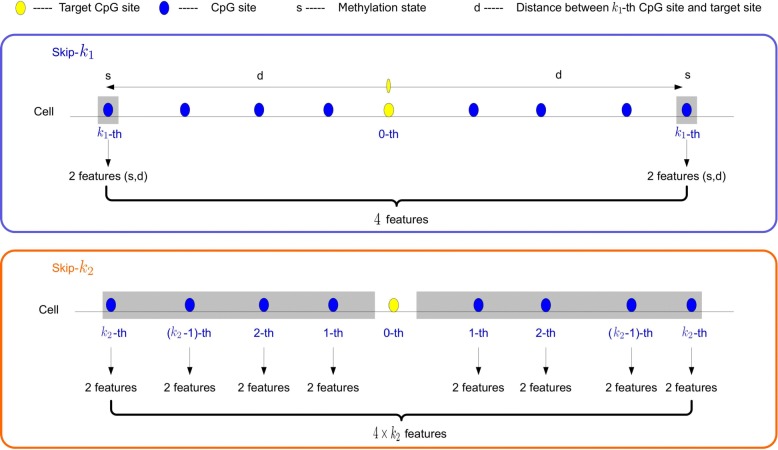
The sketch map of feature $\mathcal {F}$ and feature $\mathcal {D}$

Finally, we extracted 4*m*+8(*m*−1) features to solve the problem of multi-cell methylation identification. The sketch maps of feature $\mathcal {F}$ and feature $\mathcal {D}$ are shown in Fig. 3. All features are described in detail in Additional file 3.

### LightGBM model

**Fig. 4:**
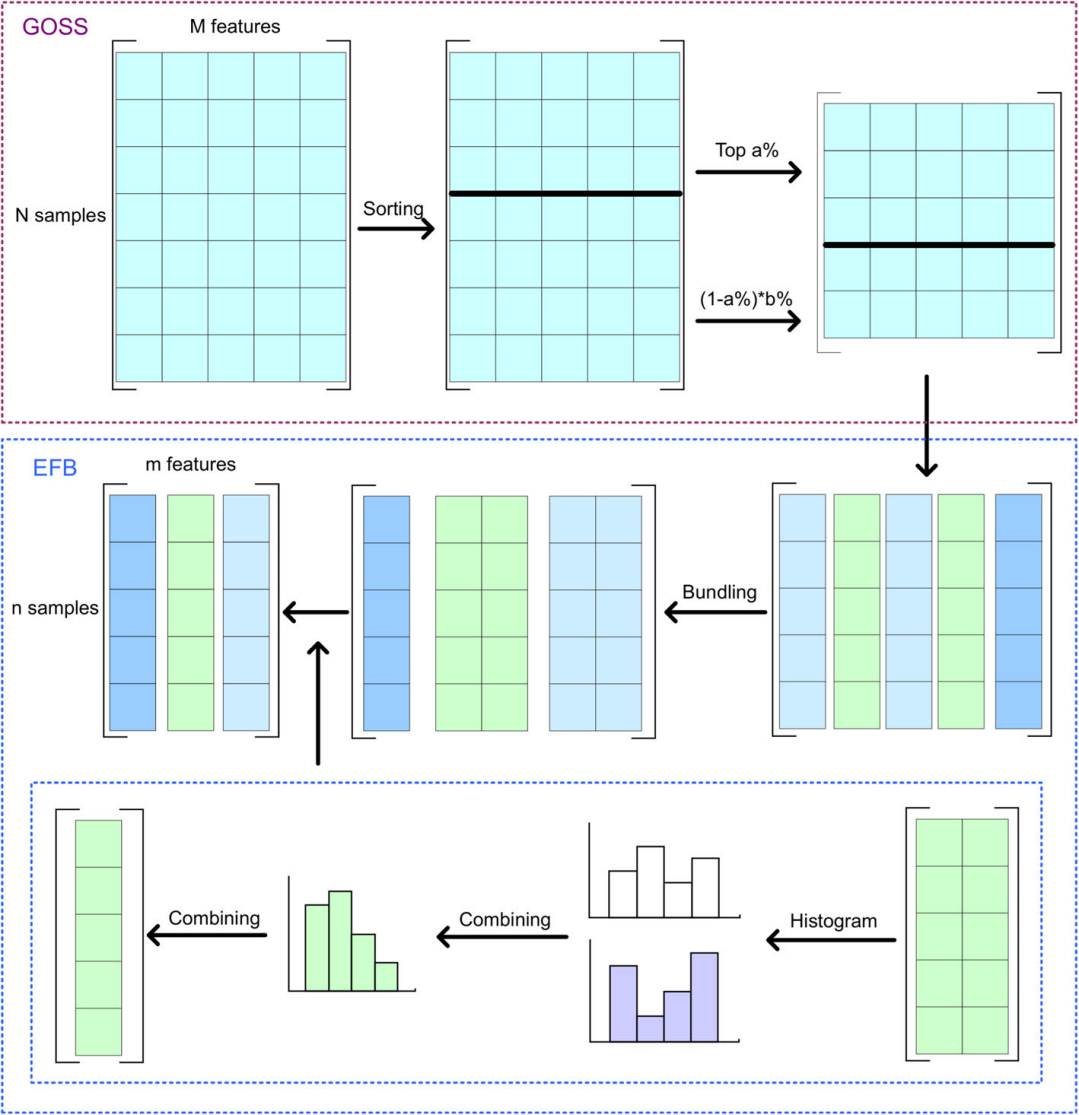
The flow chart of GOSS and EFB. **(1)** **GOSS** was used to reduce the number of samples. First, we sorted all data samples according to the gradient values. Then, the top *a%* samples were extracted and *b%* of the remaining samples were randomly selected. **(2)** **EFB** was used to reduce the number of features. First, we bundle multiple sparse features into one set and then combined a set into one feature with the help pf a histogram

In this paper, we used the LightGBM method [36] to distinguish the states of various CpG sites. The model uses a novel gradient boosting decision tree (GBDT) algorithm, including gradient-based one-side sampling (GOSS) to extract relatively small number of samples according to gradient values and exclusive feature bundling (EFB) to reduce the number of features. The GOSS and EFB approaches are described in Fig. 4.

#### Sample selection

For the GOSS approach, we first sorted all the data samples according to the gradient values. Then, the top *a%* samples were drawn and *b%* of the remaining samples were randomly selected. Finally, the two parts were combined for further analysis. Following the GOSS process, the dataset samples were reduced.

#### Feature merging

The EFB method was mainly used to handle sparse feature sets. First, we constructed a graph based on the number of non-zero values in each feature. The feature *F*_*i*_ was marked as a node in the graph and the connection weight of two features *F*_*i*_ and *F*_*j*_ was calculated as shown below:

7 $$  {w}_{i,j}= \frac{N_{i,j}}{L}  $$

where *L* represents the number of samples, and *N*_*i,j*_ represents the total number of *F*_*i*_ and *F*_*j*_ not equal to 0 at the same time.

Next, we sorted features according to the degree value in the graph, and feature with the largest degree value was used as an initial set. We defined *d* as the maximum conflict value for a feature set. Each feature was included in the existing set when the total connection weight was less than *d*. Otherwise, this feature was included as a new set when the total connection weight in all sets was greater than *d*.

Finally, we obtained a composite feature for each set using the histogram method, including multiple feature information. As the number of features decreased, the time complexity of the training process also decreased.

#### Training model

The input training set was recorded as *D* = {(*x*_1_,*y*_1_),(*x*_2_,*y*_2_),…,(*x*_*m*_,*y*_*m*_)}, where *m* is the number of samples, *x*_*m*_ is the features of *m*-th sample and *y*_*m*_ is the real output of *m*-th sample. We defined the number of trees as *T*. The loss function was denoted as *L*(*y,c*), where *y* represents the expected output and *c* is the real output.

First, we used a weak classifier *f*_0_(*x*). Before training the *t*-th tree, the gradient of each sample was calculated separately as shown below:

8 $$  g_{t}(x_{i}) = -\left[\frac{\partial{L(y_{i},f(x_{i}))}}{\partial{f(x_{i})}}\right]_{f(x)=F_{t-1}(x)} ~~~i=1,2\ldots{m}  $$

where *g*_*t*_(*x*_*i*_) represents the gradient value of the *i*-th sample inputted into the *t*-th tree and *F*_*t*−1_(*x*) represents the strong classifier of the linear combination of *t*-1 weak classifiers. Gradient values were used to train the *t*-th tree, and the learning model equation is denoted as follows:

9 $$  w^{\ast}_{t} = \arg \min \limits_{w}\sum\limits_{i=1}^{m}L(g_{t}(x_{i}),h_{t}(x_{i},w_{t}))  $$

where *w*_*t*_ represents the parameter in the training tree process, and $w^{\ast }_{t}$ represents the value of the *w*_*t*_ when the loss function takes the minimum.

Next, we solve the coefficient as shown below:

10 $$  {\rho_{t}}^{\ast} = \arg \min \limits_{\rho_{t}} \sum\limits_{i=1}^{m}L\left(y_{i},F_{t-1}(x_{i})+{\rho_{t}}h_{t}\left(x_{i},w^{\ast}_{t}\right)\right)^{2}  $$

If *f*_*t*_(*x*)=*ρ*_*t*_^∗^*h*_*t*_(*x*_*i*_,*w*^∗^), the model becomes updated as shown below:

11 $$  F_{t}(x)=F_{t-1}(x)+f_{t}(x)  $$

Finally, the output model was summarized as follows:

12 $$  F(x)=f_{0}(x)+\sum\limits_{t=1}^{T} f_{t}(x)  $$

In this work, we examined whether a CpG site was covered by the reads which were mostly methylated in the raw files. If this was true, then the CpG site was considered to be a methylation site and the state of site was denoted as 1. If a CpG site was covered by the reads which are mostly unmethylated, then the site was considered as unmethylation site and the state of CpG site was denoted as 0. Therefore, the methylation recognition is a dual classification problem. Our method utilized (259+4*m*+8(*m*−1))-*d* features to train the LightGBM classifier and predict the states of new CpG sites.

## Results

In this section, we analyzed the performance of our method from different aspects. First, we analyzed the skip- *k*_1_ and skip- *k*_2_ methods using three datasets. Second, we examined the effectiveness of our positional feature extraction approach. Third, we analyzed the performance of our feature extraction method via comparison with other two methods. Fourth, we analyzed the importance score of each feature to select the most important features. Fifth, we compared the LightGBM with four other classifiers to demonstrate which one has the most accurate performance. Sixth, we compared our LightCpG model with two other methods of methylation recognition using two databases. Finally, we compared the running time of five machine learning methods, including the LightCpG, to evaluate which method was the most efficient.

### Evaluation criteria

To establish the evaluation criteria for the prediction, we took eight mathematical measurements, including Acc, area under curve (AUC), area under the precision-recall curve (AUPR), Fscore, precision, Matthews correlation coefficient (MCC), sensitivity (SE), and specificity (SP). The formulas for each of the parameters are summarized by the following equations:

13 $$  {}Acc=\frac{TP+TN}{TP+FP+TN+FN}  $$

14 $$  {}Precision=\frac{TP}{TP+FP}  $$

15 $$  {}SE=\frac{TP}{TP+FN}  $$

16 $$  {}SP=\frac{TN}{TN+FP}  $$

17 $$  {}Fscore=2{\times}\frac{Precision{\times}SE}{Precision+SE}  $$

18 $$ {\begin{aligned}  {}MCC=\frac{TP{\times}TN-FP{\times}FN}{\sqrt{(TP+FP){\times}(TN+FN){\times}(TP+FN){\times}(TN+FP)}} \end{aligned}}  $$

where *TP* represents the number of the true methylation state, *TN* represents the number of the true unmethylation state, *FP* represents the number of false methylation state, and *FN* represents the number of false unmethylation state.

These evaluation indicators are often used to evaluate the performance of the classifier, following which they can quantify the performance from different perspectives. AUC is the area present under the receiver operating characteristic (ROC) curve. The ROC curve is created by plotting true positive rate against false positive rate at various threshold settings. Here, we used AUC as the main criterion. We often encountered unbalanced DNA methylation datasets, which required us to use the AUPR measurement. Specifically, AUPR is the area present under the curve that is created by plotting precision against recall at various threshold settings. When dealing with unbalanced datasets, AUPR was better at evaluating the performance of the classifier.

### Feature analysis

#### Analysis of parameter *k*

**Fig. 5:**
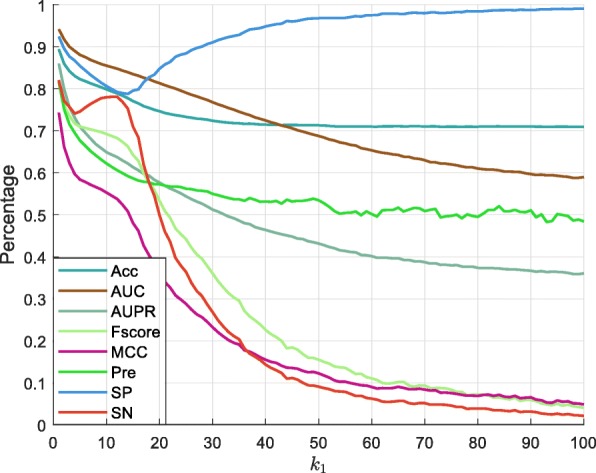
The performance of different *k*_1_ values on the GM12878 dataset

**Fig. 6:**
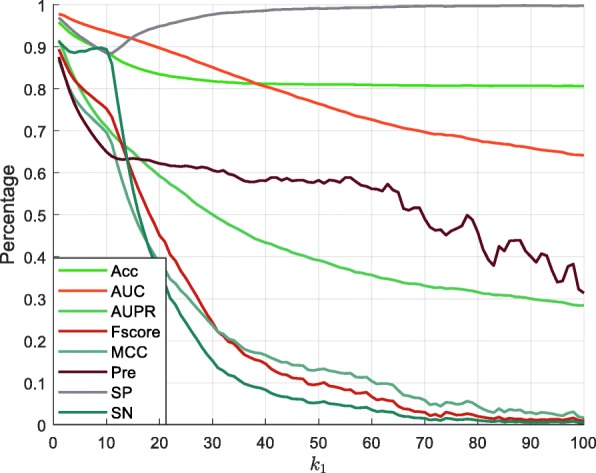
The performance of different *k*_1_ values on the heart left ventricle dataset

In this section, we analyzed the value of parameter *k*_1_ using two datasets (heart left ventricle and GM12878) in single cells. Heart left ventricle dataset was composed of 68,129 methylation sites and 280,683 unmethylation sites for training, with 56,691 methylation sites and 237,758 unmethylation sites available for testing. GM12878 contained 113,059 methylation sites and 276,808 unmethylation sites for training, with 95,348 methylation sites and 233,016 unmethylation sites available for testing. The CpG site was defined as 1 if the site was methylated, otherwise it was assigned 0. We ranged the value of parameter *k*_1_ from 1 to 100 with a step length of 1. We trained the RF model with 500 trees and the results are summarized in Figs. 5 and 6 (details available in Additional file 4. The data indicate that most of the examined mathematical measurements gradually decreased with increasing distance in both datasets. This performance revealed that the correlation between two CpG sites became weaker when the distance between them increased, so *k*_1_ was assigned a value of 1.

**Fig. 7:**
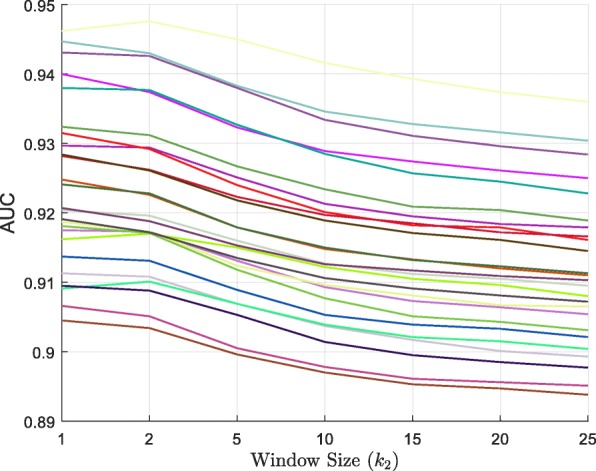
The performance of different *k*_2_ values on the HCCs dataset

Moreover, we analyzed the state of the CpG site using its upstream and downstream CpG sites in all cells. We utilized the 25 cells of human HCCs dataset in the multi-cells to construct a training set and a test set, as shown in Additional file 1. We extracted the *k*_2_ from the nearest CpG sites from upstream and downstream of the target CpG site in all cells. We then assigned the values of parameter *k*_2_ as 1, 2, 5, 10, 15, 20, and 25. The RF model was constructed for each cell to analyze the performance of different *k*_2_ values (Fig. 7 with additional information available in Additional file 5). Data presented in Fig. 7 indicate that the value of AUC decreased with the increasing value of *k*_2_. In 22 out of the 25 cells, the AUC value for *k*_2_=1 was greater than that for *k*_2_=2. Therefore, the value of *k*_2_ was set as 1.

#### Analysis of position feature

To analyze the distribution of CpG sites at identical positions in different cells, we examined chromosomes 1-12 from the 25 cells of the human HCCs dataset (Table 1). The results suggest that the methylation states of the 56.21*%* CpG sites were known at the same position in at least two cells, while the methylation states of the 14.22*%* CpG sites were identical at the same position in at least in two cells. Moreover, the 0.63*%* CpG sites had the same methylation states in specific sites where methylation state was known. For proper analysis, it is important to refer to the methylation states at the same position in different cells. For the 25 cells of the human HCCs dataset, we extracted (4×25+8×24) positional features to address the multi-cell methylation identification issue.

**Table 1:** The distribution of CpG sites with known methylation status in different cells on the HCCs dataset

Chr	*N*	*N*_1_	*P*_1_(*%*)	*N*_2_	*P*_2_(*%*)	*N*_3_	*P*_3_(*%*)
1	245976	145401	59.11	39278	15.97	1571	0.64
2	161199	87074	54.02	20338	12.62	980	0.61
3	110750	61484	55.52	14919	13.47	736	0.66
4	79228	34884	44.03	6879	8.68	407	0.51
5	102061	53635	52.55	10876	10.66	526	0.52
6	118936	67662	56.89	15998	13.45	748	0.63
7	168052	105917	63.03	32047	19.07	1318	0.78
8	99288	56661	57.07	13249	13.34	572	0.58
9	129180	77692	60.14	22570	17.47	1010	0.78
10	105721	56237	53.19	13081	12.37	542	0.51
11	124461	70626	56.75	17566	14.11	749	0.6
12	96267	49025	50.93	12301	12.78	625	0.65
Total	1541119	866298	56.21	219102	14.22	9784	0.63

^1^Chr represents the chromosome ID

^2^*N* represents the total number of known CpG sites

^3^ *N*_1_ represents the number of sites at the same position at least in two cells

^4^ *P*_1_(*%*) represents the proportion of *N*_1_ in all sites

^5^ *N*_2_ represents the number of sites with same states at the same position in at least two cells

^6^ *P*_2_(*%*) represents the proportion of *N*_2_ in all sites

^7^ *N*_3_ represents the number of sites with the same states in specific sites where methylation state was known

^8^ *P*_3_(*%*) represents the proportion of *N*_3_ in all sites

#### Performance of different features

Our method considers sequence features, structural features and positional features. We used RF classifier to analyze the performance of each feature in the 25 cells of the human HCCs dataset (Table 2). Detailed information on each evaluation criteria can be found in Additional file 6. Based on the data presented in Table 2, the ACC, MCC, and AUC values of the sequence features were 76.72*%*,29.23*%* and 0.7749, respectively. Using both sequence and structural features, the ACC, MCC, and AUC values were 78.73*%*,37.84*%* and 0.8285, respectively. Incorporating all features in the model yielded the best results where the ACC, MCC and AUC values were 90.48*%*,73.86*%* and 0.9438, respectively. These results suggest that the positional features can significantly improve the performance of our method.

**Table 2:** The performance of different features

Feature	No.	Acc(%)	AUC	AUPR	Fscore(%)	MCC(%)	Pre(%)	SP(%)	SE(%)
Seq	84	76.72	0.7749	0.5280	40.49	29.23	56.77	91.12	32.56
Str	175	77.48	0.8154	0.5468	48.68	35.17	56.99	88.70	43.00
Pos	292	90.35	0.9398	0.8587	79.56	73.49	85.06	**95.46**	74.80
Seq+Str	259	78.73	0.8285	0.5956	49.47	37.84	61.48	90.32	42.65
All	551	**90.48**	**0.9438**	**0.8597**	**79.89**	**73.86**	**85.11**	95.43	**75.39**

^1^Seq represents the sequence features

^2^Str represents the structural features

^3^Pos represents the positional features

^4^The boldface is the best value in the column

#### Performance of feature selection

**Fig. 8:**
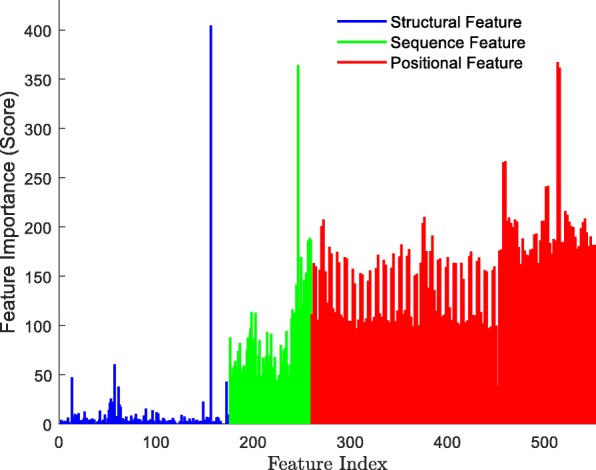
The importance score for all features on the HCCs dataset

Each feature exerts a different impact on the performance of the CpG site recognition. To examine the importance of each feature, we set up an experiment to score each feature using the LightGBM model. The importance score is the sum of the number of times each feature participates in node splitting when building a tree model. If the feature participates in the node splitting many times, the importance score will be higher. We used specific steps in the experiment with the 25 cells of the human HCCs dataset. The first step was to train 25 LightGBM models using the 25 cell training sets and extract the importance score for each feature. In the second step, the importance score of each feature in the 25 cells was added and the final statistical results were presented in Fig. 8. It is critical to note that for our method the positional features were the most important among all available features.

**Fig. 9:**
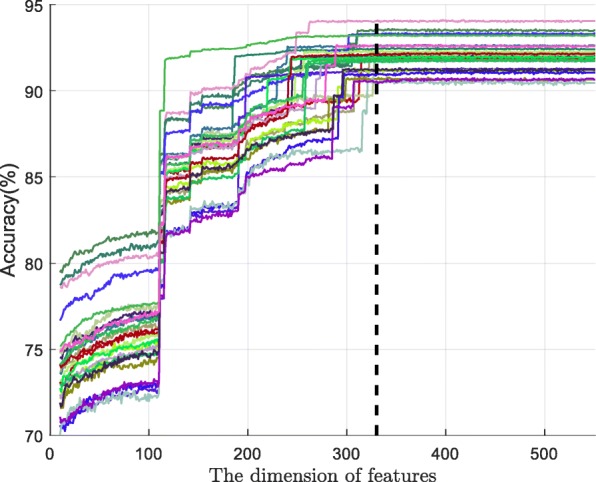
The trends of accuracy on the feature dimension in all cells on the HCCs dataset

In addition, we obtained different features according to the importance score from top 10 to top 551 in 25 cells, and trained the LightGBM model separately. The accuracy of different dimension features is shown in Fig. 9, where the *x*-axis is the dimension of the features and *y*-axis is the accuracy of the prediction. The dimension of all features was equal to 551. When the feature dimension was lower than 110, the Acc value increased steadily. In the 110 - 115 dimension range, the Acc value for the 25 cells increased rapidly. The five features from the top 110-115 dimensions were four positional features and the frequency of the combination of Adenine and Cytosine (AC) in the DNA sequence. Beyond the top 115 dimensions, the value of Acc continued to increase at a fast rate. When the dimension value reached 320, the accuracy reached the maximum in the 25 cells. Among the top 320 dimensions, we found that there were 233 positional features, eight structural features and 79 sequence features. The eight structural features included the constraint score, CGIs, histone H3 lysine 9 acetylation (H3K9ac), CGIs shelf, CCNT2 (Cyclin T2), iHS, CGIs shore, and HMGN3 (High mobility group nucleosome-binding domain-containing protein 3). The H3K9ac acetylation is very important and it can be easily silenced during DNA methylation. Some studies suggest that hypomethylated DNA is preferentially bound by the HMGN3 protein [52]. With more than 320 dimensions in our model, we determined the accuracy to be stable. The last 232 features included 167 structural features, five sequence features and 60 positional features. Overall, the accuracy of the LightGBM did not decrease with the increasing dimension but remained stable, indicating that LightGBM had a very stable performance.

### Comparison of different feature extraction methods

**Fig. 10:**
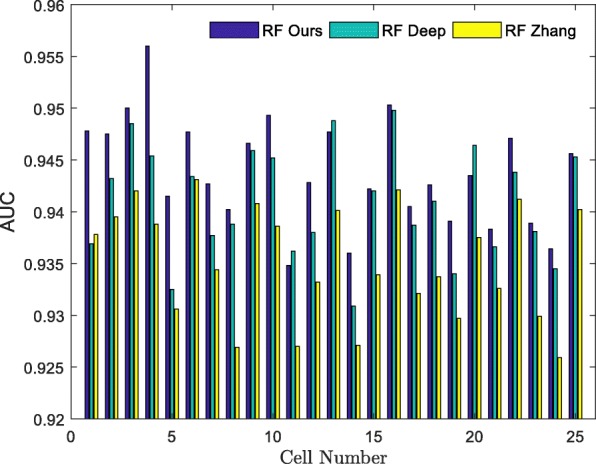
The AUCs of different feature extraction methods analyzed using the HCCs dataset. **RF** **Ours** uses ours features to train the RF model. **RF** **Deep** uses DeepCpG features to train the RF model. **RF** **Zhang** uses Zhang’s features to train the RF model

**Fig. 11:**
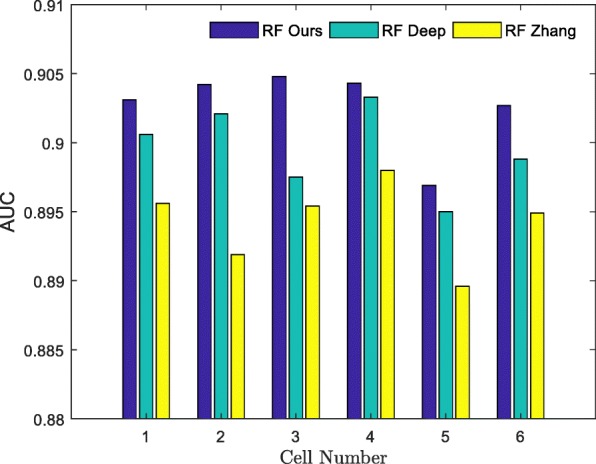
The AUCs of different feature extraction methods in the HepG2 dataset. **RF** **Ours** uses ours features to train the RF model. **RF** **Deep** uses DeepCpG features to train the RF model. **RF** **Zhang** uses Zhang’s features to train the RF model

To evaluate the performance of our method, we applied it to the human HCCs and human HepG2 datasets and compared it with the DeepCpG [35], and another method proposed by Zhang et al. [1], where RF was used as a benchmark classifier. The DeepCpG method examined 25 CpG sites upstream and downstream of the known CpG sites and used their methylation states and distances to train the GRU network model. Zhang’s method used four aspects to train the RF classifier: genomic position feature, DNA sequence properties, cis-regulatory elements (CREs) and the states and distances between the neighboring CpG sites. Figure 10 shows the AUC values of different feature extraction methods in the 25 cells in the human HCCs dataset, where the *x*-axis represents the cell number and the *y*-axis represents the predicted AUC value. Detailed information on other evaluation indicators in each cell can be found in Additional file 7 and Additional file 8. The AUC value for our method was the highest one among the 22 cells. In the remaining three cells, the difference between the AUC value for our method and that of the DeepCpG method was very small. These results suggest that our feature extraction method is likely more significant compared to the two other methods. To further demonstrate superiority and significance of our method in the feature extraction we analyzed the feature extraction method using the HepG2 dataset. Data presented in Fig. 11 indicate that the AUC value of our method for the feature extraction was the most significant in all the six cells compared to other methods.

### Comparison of different classifiers

To explore the performance of the LightGBM classifier, we compared various classifiers using the human HCCs dataset. Since the LightGBM classifier improved the Gradient Boost Decision Tree (GBDT) algorithm in terms of sample selection and feature mergence, we used the GBDT algorithm for comparison. Similarly, the XGBoost classifier [53] is also based on the GBDT algorithm, with additional improvements. Because the reference indicator is completely redefined when the tree leaf nodes split, the significant performance of XGBoost has been shown in many previous studies [54, 55]. Then, the GBDT became an integrated tree model. One of its characteristics is the nonparallel training process, which uses the gradient of the previous tree as the input for the next tree. In addition, since RF is a very stable integrated tree model, we also used it as a comparator in our analysis. The DeepCpG model has an outstanding performance due to the use of the deep learning method, we used the Fully Connected Neural Network for comparison as well. The comparative results are summarized in Table 3, where each evaluation indicator represents the average value of all cells. Detailed information on each evaluation indicator for each cell can be found in Additional file 7 and Additional file 8. Based on the data presented in Table 3, in the HCCs dataset, the Acc value of LightGBM was higher compared to other classifiers, improving by at least 0.37*%*- 1.79*%*. The AUC value of the LightGBM was higher compared to other classifiers, improving by at least 0.0062-0.0214. The Fscore, MCC, SP and SN values of the LightGBM classifier were 84.66*%*,78.97*%*,93.73*%* and 86.84*%*, respectively. The distribution of the evaluation criteria in the HepG2 dataset in each classifier was consistent with the distribution observed in the HCCs dataset. The experimental results indicate that the LightGBM classifier was more suitable for the most efficient CpG site recognition.

**Table 3:** The comparison of different classifiers

Data set	Classifier	Acc(%)	AUC	Fscore(%)	MCC(%)	SP(%)	SE(%)
HCCs	RF	90.48	0.9438	79.89	73.86	**95.43**	75.39
	GBDT	91.69	0.9538	83.11	77.58	95.11	81.31
	XGBoost	90.74	0.9554	82.89	76.73	91.23	**88.94**
	**LightGBM**	**92.06**	**0.9616**	**84.66**	**78.97**	93.73	86.84
	FCNN	90.27	0.9402	80.16	73.77	94.24	78.14
HepG2	RF	82.46	0.9027	78.98	63.92	84.94	**78.93**
	GBDT	81.80	0.8990	78.34	62.63	83.92	78.80
	XGBoost	79.42	0.9131	79.09	62.39	**93.14**	69.53
	**LightGBM**	**83.20**	**0.9213**	**81.73**	**67.36**	89.96	78.32
	FCNN	80.97	0.8841	76.76	60.70	84.93	75.35

^1^RF [28] is an ensemble learning model that uses the idea of bagging and the random selection of features to avoid data over-fitting

^2^GBDT [60] is a non-parallel model that uses the gradient from previous tree as the input for the next tree

^3^XGBoost [53] is an improved GBDT algorithm. The reference indicator of XGBoost is completely redefined when the tree leaf nodes split

^4^LightGBM [36] is based on the GBDT algorithm and employs sample selection and feature mergence to reduce the running time

^5^FCNN represents the Fully Connected Neural Network

^6^The boldface is the best value in the column

### Performance evaluation using different datasets

**Fig. 12:**
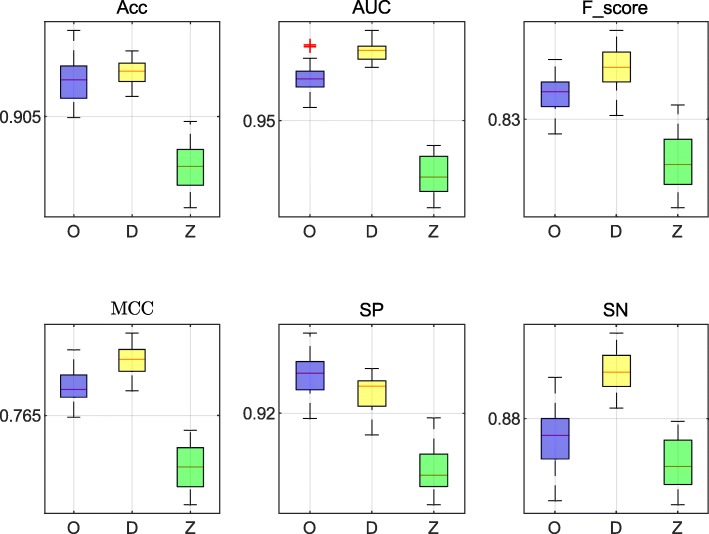
The distribution of the evaluation values using the HCCs dataset. **O** represents our method, **D** represents the DeepCpG method, and **Z** represents the method of RF Zhang

**Fig. 13:**
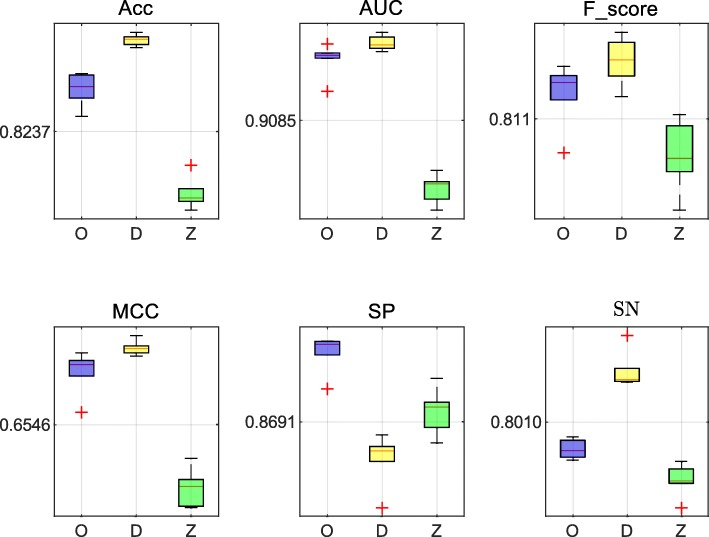
The distribution of the evaluation values using the HepG2 dataset. **O** represents our method, **D** represents the DeepCpG method, and **Z** represents the method of RF Zhang

For the HCCs dataset and the HepG2 dataset, three methods achieved excellent performance. The data presented in Tables 4 and 5 list the values of best evaluation and average evaluation in all cells, respectively. In Table 4, we first select the best performance cell by the highest ACC. Then, we list all the evaluation criteria on the cell. Results presented in Table 4 indicate that the performance of our method was similar to the other two methods. In the HepG2 dataset, the AUC value of our method was 0.9246, while DeepCpG and RF Zhang reached the values of 0.9239 and 0.8954, respectively. Moreover, our method was also better at SP (90.33*%*). In the analysis of the HCCs dataset, our method also performed better compared to the other two methods. The MCC value of our method reached 82.10*%*, which was 0.43*%* higher compared with the DeepCpG method. Moreover, the Acc and SP values were increased by 0.87*%* and 1.8*%*, respectively, and the Fscore and AUC values were almost the same as those obtained with the DeepCpG method. Additionally, based on the results presented in Table 5, our method performed well in all cells. Using the HCCs dataset, the AUC value of our method was 0.9616, which is 0.0073 lower compared to the DeepCpG method, but the RF Zhang reached the value of 0.9351. Using the HepG2 dataset, the AUC value of our method reached 0.9213. Data in Figs. 12 and 13 show the distribution of each evaluation indicator in all cells, where *O* represents our method, *D* represents the method of DeepCpG, and *Z* represents the method of RF Zhang.

**Table 4:** The comparison of different methods for the best evaluation values in all cells

Dataset	Methods	Acc(%)	AUC	Fscore(%)	MCC(%)	SP(%)	SE(%)
HCCs	**LightCpG**	**94.07**	0.9709	85.82	**82.10**	**95.74**	87.59
	DeepCpG	93.20	**0.9732**	**85.94**	81.67	93.94	**90.71**
	RF Zhang	90.29	0.9388	80.10	73.95	91.78	85.29
HepG2	**LightCpG**	83.51	**0.9246**	82.40	68.04	**90.33**	78.43
	DeepCpG	**84.08**	0.9239	**82.82**	**68.14**	82.85	**85.60**
	RF Zhang	81.25	0.8954	80.05	63.48	87.77	76.35

^1^LightCpG employs three types of features (sequence feature, structural feature and positional feature) and LightGBM [36] to identify the CpG sites

^2^DeepCpG [35] embodies the connection between various cells by using the deep learning model Gated Recurrent Network (GRU) and also extracts features from the DNA sequence by the convolutional neural network (CNN) and one additional fully connected hidden layer. Then DeepCpG uses Fully Connected Neural Network to identify CpG sites

^3^RF Zhang [1] extracts the genomic positional features, neighbor features, sequence properties and sic-regulatory elements to identify the CpG sites

^4^The boldface is the best value in the column

**Table 5:** The comparison of different methods for the average evaluation values in all cells

Dataset	Methods	Acc(%)	AUC	Fscore(%)	MCC(%)	SP	SE(%)
HCCs	**LightCpG**	92.06	0.9616	84.66	78.97	**93.73**	86.84
	DeepCpG	**92.34**	**0.9689**	**86.42**	**81.24**	92.95	**90.59**
	RF Zhang	88.41	0.9351	79.93	72.08	89.38	85.59
HepG2	**LightCpG**	83.20	0.9213	81.73	67.36	**89.96**	78.32
	DeepCpG	**84.17**	**0.9248**	**82.52**	**68.22**	85.27	**83.40**
	RF Zhang	81.16	0.8942	80.17	63.20	87.39	76.29

^1^LightCpG employs three types of features (sequence feature, structural feature and positional feature) and LightGBM [36] to identify the CpG sites

^2^DeepCpG [35] embodies the connection between various cells by using the deep learning model Gated Recurrent Network (GRU) and also extracts features from the DNA sequence by the convolutional neural network (CNN) and one additional fully connected hidden layer. Then DeepCpG uses Fully Connected Neural Network to identify CpG sites

^3^RF Zhang [1] extracts the genomic positional features, neighbor features, sequence properties and sic-regulatory elements to identify the CpG sites

^4^The boldface is the best value in the column

### Feasibility Analysis

For large-scale data, the running time is used to evaluate the feasibility of the model. For the RF classifiers, we implemented it using MATLAB scripts and executed it using a Think Station P700 computer. For the GBDT classifiers, we implemented those using Python2.7 scripts and executed them using a Think Station P700 computer. This computer has two 12-core Intel Xeon E5 CPUs and 384 GB RAM, with the CPU clock rate of 2.40GHz. For the LightGBM and XGBoost classifiers, we implemented those using Python2.7 scripts and executed them using a computer with i7-7700 CPU and 64 GB RAM. For the Fully Connected Neural Network model we used a GTX1080Ti GPU card.

In this section, we used the HCCs and the HepG2 datasets to calculate the running time of each classifier in each cell for training and listed the average time consumption of the two datasets in Table 6. The detailed information for each evaluation indicator can be found in Additional file 9. The time consumption of Fully Connected Neural Network using the HCCs dataset was an average of 4138.88 s, and the time consumption of the Fully Connected Neural Network using the HepG2 dataset was an average of 1889.55 s. The time consumption of RF and GBDT in the HCCs dataset was 252 and 20 times longer than that of the LightGBM model, respectively. The time consumption of RF and GBDT in the HepG2 dataset was 88 and 9.4 times longer than that of the LightGBM model, respectively. These results suggest that traditional machine learning method is faster than the Fully Connected Neural Network method, but both methods have similar precision.

**Table 6:** Running time of each classifier

	Dataset	FCNN	LightGBM	XGBoost	RF	GBDT
RunningTime (s)	HCCs	4138.88	8.30	5.25	2093.28	165.79
	HepG2	1889.55	5.06	2.88	450.05	57.59

^1^FCNN (Fully Connected Neural Network): the number of layers was 2, the number of nodes was the number of features, the activation function was sigmoid, the loss function was mean square error and the optimizer was RMSProp

^2^LightGBM: the number of trees was 110, the number of max depth was 5, the number of leaves was 22, learning rate was 0.04 and the number of thread was 8. Other parameters were at default values

^3^XGBoost: the number of trees was 110, the number of max depth was 7 and the number of thread was 8. Other parameters were at default values

^4^RF: the number of trees was 500 and the number of thread was 8. Other parameters were at default values

^5^GBDT: the number of trees was 110, the number of max depth was 9 and the number of thread was 8

## Discussion

The sequence features and structural features are predominantly used as the prime features in the methods focusing on the CpG site recognition. The DeepCpG model uses the connection between multiple cells to construct a deep learning model to achieve excellent accuracy. Inspired by this model, we extracted the sequence features and structural features of each site and also considered the same sites in different cells to construct the information vectors of the CpG sites. In addition, two methods of sample extraction and feature mergence were used to reduce feature redundancy and to speed up the training process.

In the beginning, we verified the correlation between the two CpG sites using the heart left ventricle and the GM12878 datasets. The experimental results indicated that as the distance between the two CpG sites increased, the majority of the evaluation indicators gradually decreased in all the datasets. In addition, we verified the correlation between the window of nearest sites and the target site, and extracted *k*_2_ sites from the upstream and downstream regions surrounding the target site. These results demonstrated that the AUC value decreased steadily as the window size increased.

Next, we calculated the correlation between the CpG sites in the 25 cells of the HCCs dataset and discovered that up to 63% of the CpG site states were known in at least two cells. Moreover, up to 19% of the CpG sites had consistent state in at least two cells. Overall, this demonstrates the effectiveness and interpretability of our positional feature approach.

We then trained the RF model using sequence features, structural features, positional features, and their combinations. Our data showed that the positional features play a major role. In addition, we used RF as a classifier for our features, DeepCpG features, and Zhang’s features, and compared the results of the three feature extraction methods. We found that when using the HCCs dataset containing 25 cells, our method achieved the best performance in 22 cells. Using the HepG2 dataset containing six cells, our method achieved the best performance in all of the cells.

Next, we used the LightGBM to rank features based on the importance scores, where we discovered that the sequence features and positional features had a positive effect on the performance of the model. We took 10-551 features with the highest importance score and established the correlation between feature dimension and accuracy. When the dimension of features was more than 320, the accuracy was stable. Therefore, feature mergence in the LightGBM model, which reduced feature redundancy, greatly improved the performance. Among the 320 features, there were eight structural features, including constraint score, CGIs, H3K9ac, CGIs shelf, CCNT2, iHS, CGIs shore and HMGN3. It demonstrates that these regions and proteins close tie with methylation sites.

Using the HCCs and the HepG2 datasets, we applied our features to the LightGBM, RF, XGBoost, GBDT, and the Fully Connected Neural Network model. Experimental results indicated that the LightGBM model performed its best in both the datasets, suggesting that LightGBM performed better in the recognition of methylation.

We then compared the LightCpG, DeepCpG, and Zhang methods using two datasets (HCCs and HepG2), and discovered that our method had excellent performance. In the cells with the best performance in the HCCs dataset, the Acc, MCC, and SP of our method were higher when compared with those of the DeepCpG model. Moreover, the AUC was only 0.0023 lower compared with that of the DeepCpG and the difference in Fscore was only 0.14*%*. In the average results from the 25 cells, the AUC of our method was 0.9616, which was lower by 0.0073, compared to that of the DeepCpG method, while RF Zhang reached the value of 0.9351. Using the HepG2 dataset, it is observed that the AUC of our method was higher compared with the DeepCpG in the cells with the best performance. On average, our method was 0.0035 lower than that in the DeepCpG in six cells. Based on both the datasets, our method was significantly better when compared with the RF Zhang method in all cells.

Finally, we used the same datasets and the same features to calculate the model training time of RF, GBDT, XGBoost, Fully Connected Neural Network and LightGBM. The experimental results indicated that Fully Connected Neural Network took the longest, which were average of 4138.88 s and 1889.55 s on the HCCs and HepG2 datasets, respectively. Our method took only average of 8.3 s and 5.06 s on two datasets, respectively. Since the LightGBM model took a long time for feature mergence, it was 3 s longer compared to XGBoost. These data indicate that our method greatly shortened the training time.

## Conclusion

In this paper, we presented the LightCpG model capable of distinguishing the CpG sites using the single-cell, whole-genome sequence data. Three types of features (positional feature, sequence feature, and structural feature) were extracted to identify the CpG sites. Two strategies (sample extraction and feature mergence) were used to reduce the training time. A comprehensive series of experiments with supporting data demonstrate that our model has a very effective feature extraction method. Two strategies significantly sped up the training of our model, making it more stable.

Our future research is focused on identifying the correlation between methylation and disease [6, 56–59], We also want to discover the direct correlation between methylation and disease by understanding intricate mechanisms of action underlying methylation. In addition, our research also focuses on the sample selection process [36], which is a complicated process based on specific functions rather than simple gradient values.

## Additional files

Additional file 1 The size of the HCCs dataset. (XLSX 14.3 kb)

Additional file 2 The size of the HepG2 dataset. (XLSX 10.8 kb)

Additional file 3 The composition of features. (XLSX 14.2 kb)

Additional file 4 The performance of different distances using the heart left ventricle and GM12878 datasets. (XLSX 32.1 kb)

Additional file 5 The performance of different windows using the HCCs dataset. (XLSX 29.5 kb)

Additional file 6 The performance of different features using the HCCs dataset. (XLSX 21.3 kb)

Additional file 7 The results of different methods using the HCCs dataset. (XLSX 35.8 kb)

Additional file 8 The results of different methods using the HepG2 dataset. (XLSX 15 kb)

Additional file 9 The running time of different classifiers. (XLSX 12.8 kb)

## Abbreviations

AUPR: Area under the precision-recall curve
BGS: Bisulfite genomic sequence
CGIs: CpG islands
CNN: Convolutional neural network
CREs: Cis-regulatory elements
Cyclin T2: CCNT2
EFB: Exclusive feature bundling
FCNN: Fully Connected Neural Network GRU: Gated Recurrent Network
GBDT: Gradient Boost Decision Tree
GOSS: Gradient-based one-side sampling
HCC: Human hepatocellular carcinoma cells
HepG2: Human heptoplastoma-derived
H3K9ac: Histone H3 lysine 9 acetylation
HRM: High-resolution melting
iHS: Integrated haplotype score
MSP: Methylation-specific PCR
MCC: Matthews correlation coefficient
PCA: Principal component analysis
RF: Random forest ROC: Receiver operating characteristic
scTrio-seq: Single-cell triple omics sequencing
RRBS: Reduced representation bisulfite sequencing
SNP: Single nucleotide polymorphisms
SVM: Support vector machine
SE: Sensitivity
SP: Specificity
scBS-seq: Single-cell bisulfite sequencing
scRRBS-seq: Single-cell reduced-representation bisulfite sequencing
TFBS: Transcription factor binding site
