# Correction to: LightCpG: a multi-view CpG sites detection on single-cell whole genome sequence data

**DOI:** 10.1186/s12864-019-5742-x

**Published:** 2019-05-13

**Authors:** Limin Jiang, Chongqing Wang, Jijun Tang, Fei Guo

**Affiliations:** 1 School of Computer Science and Technology, College of Intelligence and Computing, Tianjin University, Tianjin, China; 2 School of Chemical Engineering and Technology, Tianjin University, Tianjin, China; 3 Department of Computer Science and Engineering, University of South Carolina, Columbia, SC USA

**Correction to: BMC Genomics**

**https://doi.org/10.1186/s12864-019-5654-9**

Following the publication of this article [1], the authors reported that the images of Fig. 2 and Fig. 3 were switched during typesetting. The correct Fig. 2 and Fig. 3 are reproduced in this Correction article. The original article has also been corrected.

**Fig. 2:**
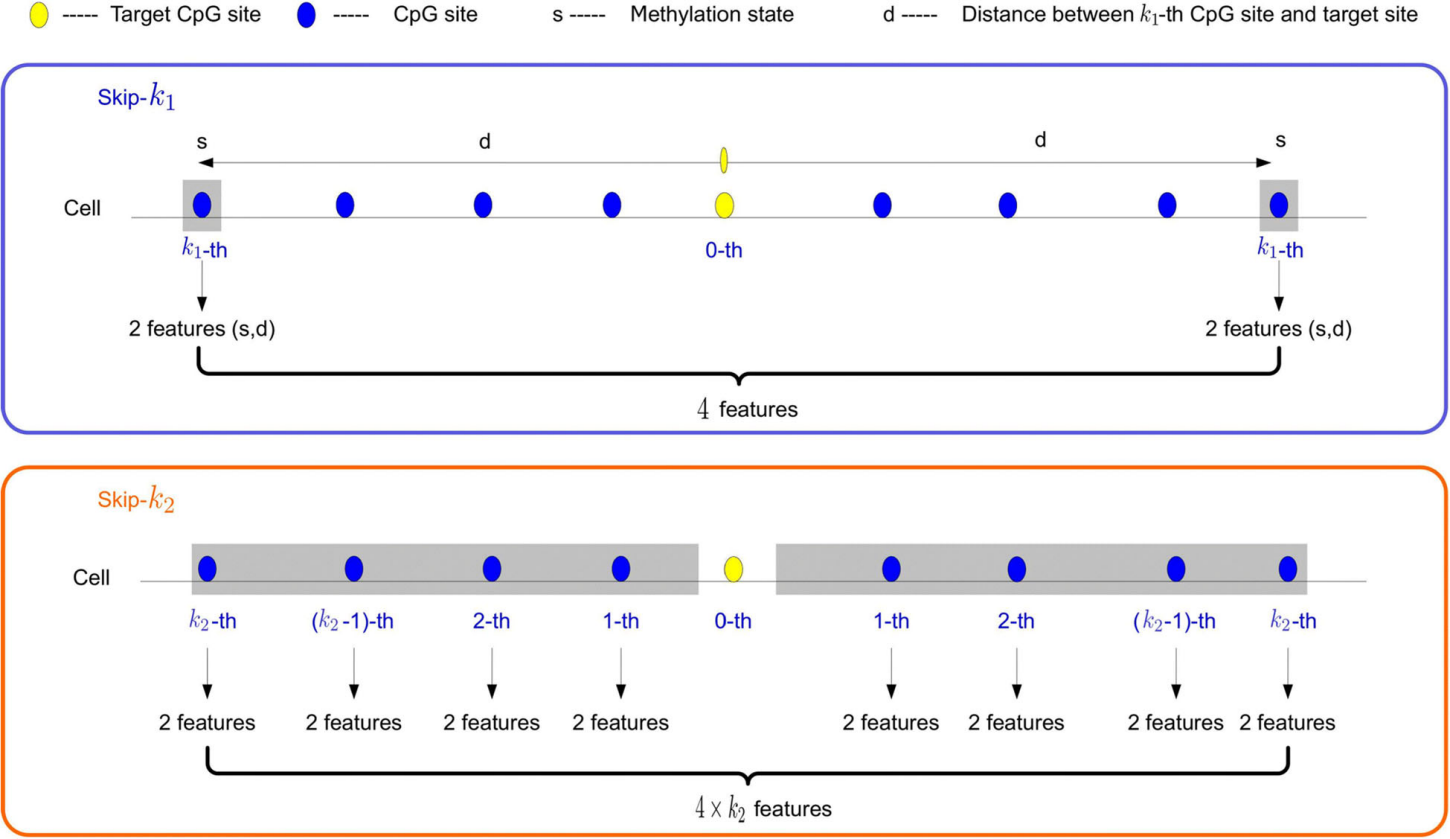
The sketch map of skip-*k* method

**Fig. 3:**
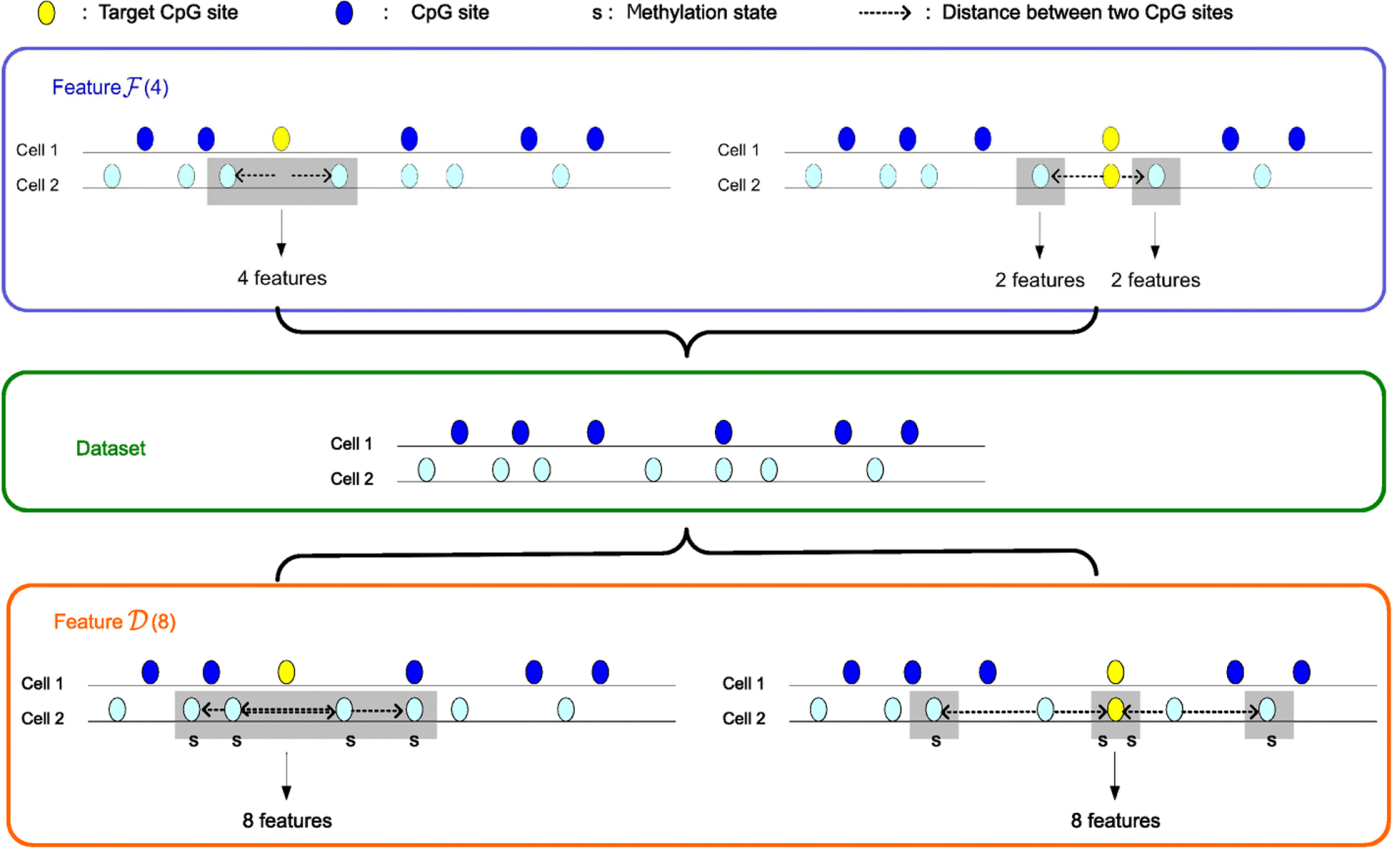
The sketch map of feature $\mathcal{F}$ and feature $\mathcal{D}$
